# Effect of Antibiotic, Phytobiotic and Probiotic Supplementation on Growth, Blood Indices and Intestine Health in Broiler Chicks Challenged with *Clostridium perfringens*

**DOI:** 10.3390/ani10030507

**Published:** 2020-03-18

**Authors:** Elsayed O. S. Hussein, Shamseldein H. Ahmed, Alaeldein M. Abudabos, Mashael R. Aljumaah, Manal M. Alkhlulaifi, Mohamed A. Nassan, Gamaleldin M. Suliman, Mohammed A. E. Naiel, Ayman A. Swelum

**Affiliations:** 1 Department of Animal Production, College of Food and Agriculture Sciences, King Saud University, P.O. Box 2460, Riyadh 11451, Saudi Arabia; gsuliman@ksu.edu.sa (G.M.S.); aswelum@ksu.edu.sa (A.A.S.); 2 Department of Basic Sciences, College of Veterinary Medicine, Sudan University of Science and Technology, P.O. Box 121, Khartoum 11123, Sudan; shamshahmed@yahoo.com; 3 Department of Botany and Microbiology, College of Science, King Saud University, P.O. Box 2455, Riyadh 11451, Saudi Arabia; maljumaah1@ksu.edu.sa (M.R.A.); manalk@ksu.edu.sa (M.M.A.); 4 Department of Pathology, Faculty of Veterinary Medicine, Zagazig University, Zagazig 44511, Egypt; moh_nassan@yahoo.com; 5 Department of Animal Production, Faculty of Agriculture, Zagazig University, Zagazig 44511, Egypt; mohammednaiel.1984@gmail.com; 6 Department of Theriogenology, Faculty of Veterinary Medicine, Zagazig University, Zagazig 44511, Egypt

**Keywords:** probiotic, phytobiotic, broiler, *C. perfringens*, histopathological, intestinal health

## Abstract

**Simple Summary:**

Necrotic enteritis is one of the most important economic issues in the poultry industry, associated with sudden death rates of up to 50%. However, there is limited information on the role of probiotics and/or phytobiotic compounds on the treatment and prevention of *Clostridium perfringens* infections in broiler chicks. This study aimed to assess the effects of probiotic compounds (Maxus, CloStat, Sangrovit Extra, CloStat + Sangrovit Extra and Gallipro Tech) on the growth performance, blood biochemistry and intestinal health of broiler chicks in vivo. The results demonstrated that the inclusion of probiotic and/or phytobiotic compounds has a positive effect on performance, blood constituents, liver histopathology, intestinal morphology and histopathology. Furthermore, a notable reduction in both lesion scores was observed when probiotics and phytobiotics alone or in combination were included in the diets.

**Abstract:**

This study evaluated the effects of feed additives on the growth, blood biochemistry and intestinal health of broiler chicks. A total of 378 of broiler chicks (Ross 308) were randomly allotted to seven groups. Chicks were fed a basal diet with 0.0 (control negative), 0.0 (control positive), 0.1, 0.5, 0.12, 0.5 + 0.12 and 0.2 g Kg^−1^ of Maxus, CloStat, Sangrovit Extra, CloStat + Sangrovit Extra and Gallipro Tech, respectively for 35 days. After 15 days, the chicks were inoculated with *Clostridium perfringens.* All feed additives were found to enhance growth performance and feed efficiency. The best feed conversion ratio was found in the Negative Control, CloStat + Sangrovit Extra and Gallipro Tect groups, respectively. A notable increase in villus length, total villus area, small intestine weight, ilium weight and total lesion score was found in chicks supplemented with *Bacillus subtilis*. Besides, the dietary inclusion of phytobiotic compounds showed potential in reducing the serum Alanine aminotransferase (ALT) concentration and increasing the glucose levels. All intestine and liver histopathological signs were reduced in chicks fed a probiotic-supplemented diet. Our findings indicate that supplementation with probiotics and phytobiotics alone or in combined form can be used to enhance performance, intestine health and blood constituents against *C. perfringens* infection in broiler chicks.

## 1. Introduction

*Clostridium perfringens* (*C. perfringens*) is a Gram-positive, anaerobic, germ-growing bacteria found within the gastrointestinal (GI) tract of several animals and in the environment [1,2] and poses an important threat to animals [3]. *C. perfringens* strains can produce up to 17 different toxins, where each set of toxins is responsible for a specific disease [4]. *C. perfringens* bacteria are responsible for several known infections in animals, including enterotoxaemia, gangrenous dermatitis and necrotic enteritis (NE), especially in poultry [5,6]. The induction of NE by *C. perfringens* in chicks can result in sudden death, with mortality rates of up to 50% [7,8]. *C. perfringens* bacteria are also responsible for subclinical infections, related to the chronic intestinal mucosa damage [9], which can have serious consequences, including decreased growth performance and weight gain and economic losses [10,11]. The cost of NE not only includes the direct loss due to the sudden death of broilers but also veterinary and scrubbing costs [12]. *C. perfringens* is generally always found in healthy chicks, even if at low levels (<10^5^ CFU/g) in the intestinal tract [9]. Several factors can create a favorable environment in the intestine for the proliferation of the bacterium and the subsequent development of disease [9]. The coccidiosis incidence is one of the most important of these factors [13,14]. However, there are also several factors related to diet that are conducive to disease, including the epithelial thickness, the incidence of non-digestible polysaccharides and the intestinal pH value [15,16,17]. Bacterial cells or spores infection can be induced in animals through the type of feed, contaminated litter or by cross-contamination with infected early-stage animals [18]. Young animals are primarily at risk due to their immature immune systems and underdeveloped intestinal flora [19]. Infected animals manifest lesions of the jejunum and ileum, while the small intestine shows a degenerated mucosa and can be swollen by gases produced by *C. perfringens* [20]. Some visible symptoms of infection include changes in behavior, less movement and diarrhea [21]. 

Several strategies have been suggested for the prevention and control of NE infection in poultry [22]. The use of feed additives for the prevention of gut diseases via enhancing the intestinal microbiota has drawn the attention of nutrition scientists in an animal in vivo studies [23]. The supplementation of diets with antibiotics is a well-known strategy against NE in broiler chicks [24]. *Avilamycin*, an oligosaccharides antibiotic, is effective against many pathogenic and Gram-positive bacteria [25]. Furthermore, it has wide in vitro bactericidal effects, especially against *C. perfringens* [25]. Paradis et al. [22] and Mwangi et al. [26] found a strong relationship between the inclusion rate of *avilamycin* in the diet and a reduction in the mortality rate, lesion scores and *C. perfringens* counts induced by NE diseases. 

Using probiotics as a food supplement improves the intestinal microbial balance of the host [27]. Probiotics cooperate with the host to enhance intestinal immunity and morphology but can also induce metabolism function, thus decreasing the risk of infection by opportunistic pathogenic bacteria [9]. Moreover, probiotic bacteria can play an antimicrobial role by producing molecules with antibacterial activities, such as bacteriocins, which can target certain pathogens and prevent the adhesion of pathogens or the excretion of pathogenic toxins [28,29]. Furthermore, beneficial bacteria can protect the host against pathogenic strains growing within the digestive tract [30]. Several studies have previously reported on the beneficial role played by certain *Bacillus* and *Lactobacillus* strains against *C. perfringens* activity in vitro [31,32].

The supplementation of broiler diets with *Bacillus* spores (*B. licheniformis*) has also been reported to result in a beneficial role played by probiotics when supplemented at high doses for extended periods compared to antibiotics [33]. After the inoculation of 20-day-old chicks with a low dosage of *C. perfringens*, the supplementation of their diet with a single dose of 1 × 10^9^ *Bacillus subtilis* mitigated the colonization and insistence of *C. perfringens*, even though *Bacillus subtilis* did not affect *C. perfringens* in vitro [34]. Similarly, Sokale et al. [35] found that the supplementation of broiler chicks with *Bacillus subtilis* alone resulted in improved production, growth performance and reduced mortality after a *C. perfringens* challenge. Therefore, supplementation with *Bacillus subtilis* can not only be used to control NE diseases but also enhances gut health in broiler chicks [36].

Despite these findings, there are no studies on the ameliorative role of other probiotics (Maxus, CloStat, Sangrovit Extra, Gallipro Tech) as feed additives alone or in combined form in terms of growth, blood biochemical parameters and gut health of *C. perfringens*-challenged broiler chicks. As such, this study investigated the beneficial effects of dietary supplementation with feed additives on the growth parameters, feed utilization, hematological profile, liver histopathology, intestine morphometrics and histopathology of broiler chicks, as well as their health and performance under disease stress.

## 2. Materials and Methods 

Experiments were performed at the Animal Production Department, College of Food Science and Agriculture Science, King Saud University. All of the protocols were performed and approved according to the experimentation guidelines of the Ethics of Animal Use in Research Committee of King Saud University (Ethical reference No: SE-19-150).

### 2.1. Feeding Trial and Regimen

For the experimental trials, 378 1-day-old broiler chicks (Ross 308) were selected and randomly allocated into seven groups (Table 1). Each group contained nine replicates, with six chicks per replicate. Upon arrival, all chicks were verified for the absence of *Salmonella* and *C. perfringens*. The broiler chicks were raised in cage pens (Dimensions of each pen were 5 × 3 × 2.5 feet) under similar managerial and hygienic conditions in an environmentally controlled poultry unit. Shed temperature was set for 1 week to 35 °C and then every week it was decreased gradually until it reached 24 °C. The lighting program followed standard ROSS TECH Lighting for Broilers [37]. Standard starter (0–15) and finisher (16–33 days) diets comprised of isocaloric and isonitrogenous ingredients were provided in mash form based on corn-soybean meal (SBM) and formulated to meet the requirements of Ross 308 broiler chicks (Table 2). 

### 2.2. Challenge Inoculum 

On day 15, all of the group except for the negative control group were challenged with *C. perfringens* (Micro Biologics, Cloud, MN, USA) at a rate of 4 × 10^8^ CFU/g via oral gavages [38]. Necropsies were carried out from the first day of infection to determine the cause of mortality. Confluent necrosis and sloughing of the intestinal epithelium were considered as signs of NE. *C. perfringens* was isolated from chicks were died because of NE. 

### 2.3. Performance Measurements

During the feeding trial period (days 0‒35), the growth performance of the broiler chicks was evaluated by recording their feed intake daily. To this end, the amount of feed rejected was subtracted from the feed offered to determine the feed intake. Also, live body weight was recorded at weekly intervals, while the final body weight and total feed consumption were recorded at the end of the feeding trial period. Body weight gain was calculated as the difference between the live body weight and the final body weight. The feed conversion ratio (FCR) was computed for each group Abudabos et al. [39] using the following formula—FCR = feed intake/weight gain. The production efficiency factor (PEF) was calculated Griffin [40] using the following formula:

PEF = (livability × live weight (kg))/(age in days × FCR) × 100.(1)

During the feeding trial, the number of deaths was counted to calculate the survival rate as the percentage of the surviving to the initial number of broilers

### 2.4. Intestine Morphometric Analysis and Lesions Score

In order to evaluate the intestinal characteristic (small and large intestines, including the ceca) and the lesions score of necrotic enteritis were randomly collected from nine broiler chicks in each group and weighed immediately after slaughtering according to Jensen et al. [41]. Before slaughter, the feed was withdrawn for six hours to ensure that the digestive tract was empty and the live body weight was determined. After slaughtering, the birds were defeathered and eviscerated. After removal, the duodenum, jejunum, ileum, caeca and small intestine were weighed and measured. The small intestine was measured between the site from which the duodenum emerges from the gizzard and the beginning of the ceca. The relative organ weight was calculated according to Arif et al. [42] formula:

the relative organ weight = (weight of organ/live weight of bird) × 100.(2)

All of the chicks were weighed before sacrifice. The gross intestinal lesions characteristic of necrotic enteritis were determined according to Long et al. [43] procedure. The lesion scores were annotated as follows: 0 = none, 1 = mild, 2 = moderate and 3 = marked (severe) [44].

The length, width and total surface area of the villus in the intestine were used as the intestinal morphometric variables [45]. 2-cm segments were dissected from the midpoint and the distal end of the small intestine of the broiler chicks. These segments were flushed with phosphate-buffered saline (PBS) (pH 7), fixed in Clark fixative for 45 min and stored in ethyl alcohol (50%). Each segment was then divided into two sections along its length (sections A and B). Section A was placed in periodic acid Schiff (PAS) reagent for 2–3 min and observed under a dissecting microscope at 100× in five randomly chosen fields of view (using a zigzag line). The types and number of different villi were recorded. Section B of each sample was allowed to stand in PAS for staining. The muscle layers were then separated from the mucosa and the rows of villi were cut in sagittal sections before being transferred onto glass slides and covered with a cover slip. These samples were examined using a microscope in the direction of the rows with an eye piece graticule at 100× [46]. The villus height (µm) and width (μm) were measured from the top of the villus to the top of the lamina propria using image capture and analysis system (Image-Pro Plus version 4.5, Media Cybernetics, Silver Spring, MD, USA). The surface area was calculated using the following formula:

(3) ¼(2 π)(VW/2)(VL)

where VW is the villus width and VL is the villus length [47]. This was repeated for 8 chicks/treatment/age in triplicate. For each chick, two segments from the medium and distal intestine were examined.

### 2.5. Blood Biochemical Measurements

At the end of the feeding trial, 3 ml of blood were obtained from the wing vein of six chicks per treatment. The blood samples were centrifuged at 3000× *g* for 10 min and the resulting serum was stored at −20 °C until further analysis. The serum indices, total protein (TP) and albumin values were determined using the method described by Reinhold [48]. The globulin levels were calculated as the difference between the total protein and albumin levels. Cholesterol [49], glucose, triglyceride [50] and serum enzyme (ALT and AST) activities were determined spectrophotometrically [51,52] using an enzymatic kit (Witte kamp 30. D-30163; MDI Europa GmbH, Hannover, Germany). 

### 2.6. Histopathological Examination

For histopathological examination, the tow broiler chicks from each replicate were sacrificed at the end of the feeding trial. The Eighteen longitudinal sections of the intestines (from three parts, anterior, mid and posterior) and liver from each treatment were cut and fixed overnight in 10% buffered neutral formalin solution. The fixed tissues were processed and stained with hematoxylin and eosin (HE), as described by Naiel et al. [53]. Then, five μm stained sections were observed and analyzed histopathologically, as reported by Bancroft and Gamble [54]. 

### 2.7. Statistical Analysis

The collected data were statistically analyzed using one-way analysis of variance (ANOVA). Significant differences were determined using Duncan’s Multiple Range [55]. All analyses were performed using SPSS software (version 14) (Chicago, IL, USA). The results are presented as the mean ± standard error (SE). 

## 3. Results

### 3.1. Growth Performance and Feed Efficiency

The effects of diet supplementation on growth performance parameters, such as feed intake (FI), body weight gain (BWG), feed conversion ratio (FCR) and production efficiency factor (PEF) in 0–35-day-old broiler chicks challenged with *C. perfringens* are presented in Table 3. The Final Body Weight (FBW) and Body weight gain (BWG) were found to increase significantly (*p* < 0.01) in the entire supplemented group compared with the positive control group. Concerning FI, none of the probiotic bacteria were found to affect this parameter after the feeding trial. Conversely, the FCR was found to decrease significantly (*p* < 0.001) in the treated groups compared to the positive control group. The lowest values were recorded in the NC, CL + S and G groups (1.60, 1.72 and 1.78, respectively). Concerning PEF, all experimental additives demonstrated highly significant (*p* < 0.05) PEF values compared to the positive control. Also, the percentage (%) of survival rate (SR) was increased in all of the experimental groups (Table 3). 

### 3.2. Intestinal Histomorphometric Measurements

The effects of feed supplementation on the intestine histomorphometric measurements (villus length (VL), villus width (VW), total villus area (TVA), small intestine length (SIL), small intestine weight (SIW), duodenum length (DL), duodenum weight (DW), jejunum length (JL), jejunum weight (JW), ileum length (IL), ileum weight (IW), ceca length (CL) and ceca weight (CW)) in the broiler chicks challenged with *C. perfringens* are presented in Table 4. The villus length and total villus area were significantly (*p* < 0.01) enhancement by feed supplementation and in the negative control group compared to the positive control group. By contrast, villus length did not show any significant differences between any of the groups.

The majority of the intestinal morphological factors were not affected by dietary supplementation with probiotics, apart from the small intestine weight (SIW), jejunum weight (JW), ileum weight (ILW) and intestine score lesion. Small intestine weight (SIW) was not significantly (*p* < 0.05) altered in the broiler chicks supplemented with probiotics compared to the control groups. However, supplementation with probiotic bacteria was found to enhance SIW and JW. The CL, S and CL + S mixed diets were found to improve JW compared to the M, G and PC groups. The highest values of JW were found in the negative control group. In terms of ileum weight (ILW), none of the additives were found to affect the ILW. Moreover, the highest values of ILW were observed in the negative control. Highly significant (*p* < 0.001) decreases in the lesion score were detected in all experimental groups compared to the positive control group. 

### 3.3. Serum Profile

The blood serum profiles in terms of the composition (total protein, TP; albumin, ALB; globulin, GLB; cholesterol, CHO; total glyceride, TG; glucose, Glu) and enzymatic activity (alanine amine transferase, ALT; alanine amine transferase, AST) are presented in Table 5. No significant differences (*p* > 0.05) were observed in blood composition or enzyme profiles between the different treatments in broiler chicks challenged with *C. perfringens*. However, the ALT and glucose levels were found to be significantly (*p* < 0.05) affected by diet supplementation. The levels of ALT were found to decrease significantly (*p* < 0.05) in the M, S, G and Nc groups, while the glucose concentration increased compared to the positive control and other treatments (Table 5).

### 3.4. Histopathological Examination of Intestine and Liver

**Figure 1:**
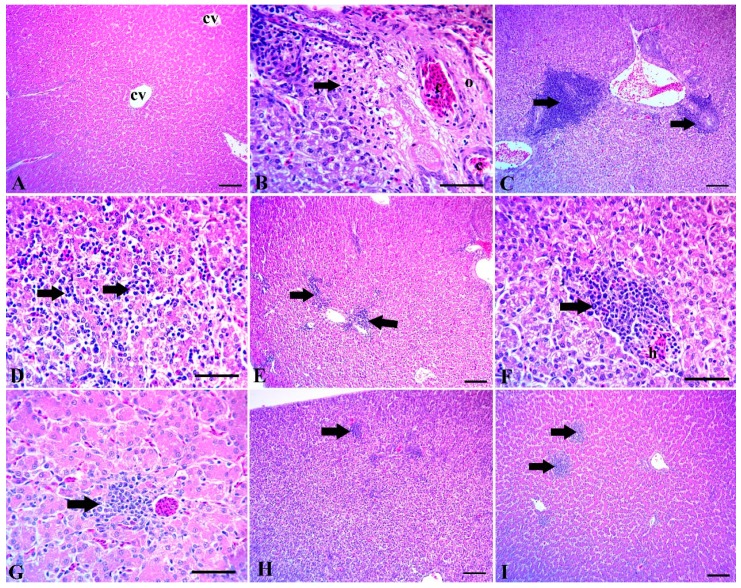
(**A**) Liver of control group without *Clostridium* challenge, showing normal tissue architecture, normal hepatic lobules, a normal central vein (cv) and hepatic sinusoids. (**B–D**) The intestine of chicks after the *Clostridium* challenge. (**B**) Congestion of portal blood vessels (c) with perivascular edema (o) and coagulative necrosis of the surrounding hepatocytes (arrow). (**C**) Numerous lymphocytic aggregations around the congested portal blood vessels. (**D**) Extensive lymphocytic aggregations among the hepatocytes (arrows). (**E–I**) Liver of chicks challenged with *Clostridium* and supplemented with: (**E**) Maxus, showing moderate scattered lymphocytic aggregations (arrows); (**F**) Clostat, showing mild hemorrhage and (**H**) focal aggregation of lymphocytes (arrow); (**G**) Sangrovit, showing mild perivascular lymphocytic aggregation (arrow); **(H)** Clostat + Sangrovit, showing mostly normal hepatic tissue, except for minute focal lymphocytic aggregations (arrow); (**I**) Gallipro, showing moderate focal lymphocytic aggregations. Scale bar = 100 µm (**A,C,E,H,I**) and 50 µm (**B,D,F,G**).

The examined liver sections of the control group showed normal tissue architecture with normal hepatic lobules, a normal central vein and normal hepatic sinusoids (Figure 1A). The liver of the challenged broiler chicks showed several histopathological signs such as portal blood vessel congestion with perivascular edema and coagulative necrosis of the surrounding hepatocytes (Figure 1B). Also, numerous lymphocytic aggregations around the congested portal blood vessels were detected (Figure 1C). Moreover, Extensive lymphocytic aggregations were detected among the hepatocytes in the *Clostridium*-challenged groups (Figure 1D). The liver of the *Clostridium*-challenged broiler chicks supplemented with Maxus reduced lymphocytic aggregations among the hepatocytes (Figure 1E). Similarly, the liver of *Clostridium*-challenge chicks supplemented with Clostat showed mild hemorrhaging and focal aggregation of lymphocytes (Figure 1F), while the liver of *Clostridium*-challenged chicks supplemented with Sangrovit showed mild perivascular lymphocytic aggregation (Figure 1G). The liver of *Clostridium*-challenged chicks supplemented with Clostat + Sangrovit showed mostly normal hepatic tissue, except for minute focal lymphocytic aggregation (Figure 1H). Lastly, the liver of *Clostridium*-challenged chicks supplemented with Gallipro showed moderate focal lymphocytic aggregations (Figure 1I).

**Figure 2:**
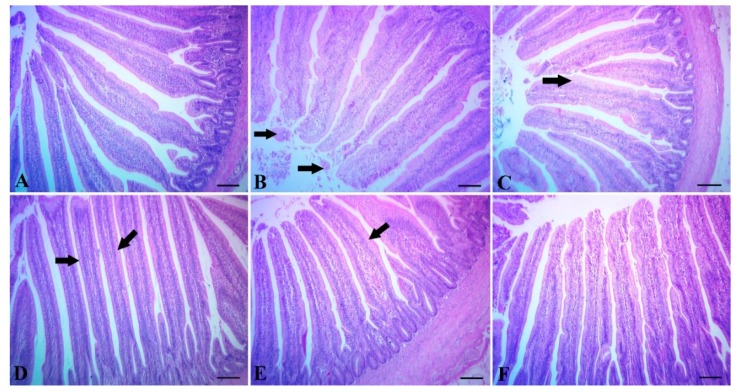
(**A**) Intestine of the control group without *Clostridium* challenge, showing normal tissue architecture. (**B**) The intestine of chicks supplemented with Maxus showing mild desquamation of villous epithelium (arrow). (**C**) The intestine of chicks supplemented with Clostat, the columnar epithelium lining the villi into goblet cells (arrow). (**D**) The intestine of chicks supplemented with Sangrovit, showing moderate metaplasia of the columnar epithelium lining the villi into goblet cells (arrow). (**E**) The intestine of chicks supplemented with Clostat + Sangrovit, showing mostly normal villi, except for mild metaplasia of the columnar epithelium lining the villi into goblet cells (arrow). (**F**) The intestine of chicks supplemented with Gallipro, showing normal tissue architecture with normal intestinal villi. Scale bar = 100 µm.

The intestine of the control group showed normal tissue architecture with normal intestinal villi (Figure 2A). Chicks supplemented with Maxus showed mild desquamation of the villous epithelium (Figure 2B), while chicks supplemented with Clostat or Sangrovit showed moderate metaplasia of the columnar epithelium lining the villi into goblet cells (Figure 2C,D). Chicks supplemented with Clostat + Sangrovit showed mostly normal villi, except for mild metaplasia of the columnar epithelium lining the villi into goblet cells (Figure 2E). Lastly, chicks supplemented with Gallipro showed normal tissue architecture with normal intestinal villi (Figure 2F). 

**Figure 3:**
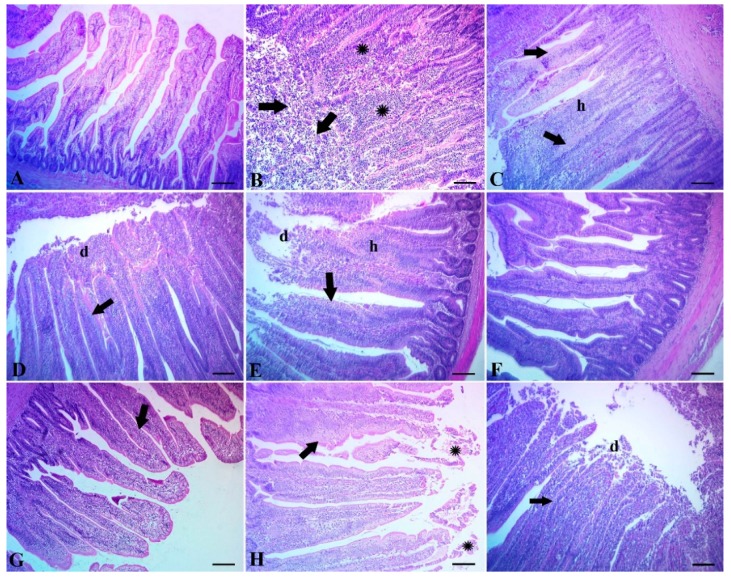
(**A**) Intestine of the control group without *Clostridium* challenge, showing normal tissue architecture with normal intestinal villi. (**B**) The intestine of chicks subjected to *Clostridium* challenge, showing an extensive degeneration and necrosis of the intestinal villi (arrows) with extensive round cell aggregations **(∗)**. (**C**) The intestine of *Clostridium*-challenged chicks showing extensive hyperplasia of the villous epithelium **(h)** with thickening and shortening of villi, together with metaplasia into goblet cells (arrows). (**D**) The intestine of *Clostridium*-challenged chicks, showing an extensive degeneration of the villous epithelium **(d)** with metaplasia of the columnar epithelium into goblet cells (arrow). (**E–I**) Intestine of chicks challenged with *Clostridium*) and supplemented with: (**E** Maxus, showing extensive hyperplasia of intestinal epithelium **(h)**, metaplasia into goblet cells (arrow) and moderate desquamation of superficial epithelium **(d)**; (**F**) Clostat, showing a regeneration of the intestinal epithelium with normal tissue architecture; (**G**) Sangrovit, showing a normal tissue architecture with moderate metaplasia of the intestinal epithelium into goblet cells (arrow); (**H**) Clostat + Sangrovit, showing a sloughing of the superficial epithelium **(∗)** with moderate metaplasia into goblet cells (arrow); (**I**) Gallipro, showing a severe desquamation of the superficial epithelium **(d)** and metaplasia into goblet cells (arrow). Scale bar = 100 µm.

The intestine of the control group without the *Clostridium* challenge showed normal tissue architecture with normal intestinal villi (Figure 3A). Broiler chicks subjected to *Clostridium* challenge showed extensive degeneration and necrosis of the intestinal villi with extensive round cell aggregations (Figure 3B). The same groups showed extensive hyperplasia of the villous epithelium with thickening and shortening of villi, together with metaplasia into goblet cells (Figure 3C). Extensive degeneration of the villous epithelium, with metaplasia of the columnar epithelium into goblet cells, was also detected (Figure 3D). The intestine of chicks challenged with *Clostridium* and supplemented with Maxus showed extensive hyperplasia of the intestinal epithelium, metaplasia into goblet cells and moderate desquamation of the superficial epithelium (Figure 3E). The intestine of chicks challenged with *Clostridium* and supplemented with Clostat showed regeneration of the intestinal epithelium with a normal tissue architecture (Figure 3F), while the intestine of chicks supplemented with Sangrovit showed a normal tissue architecture with moderate metaplasia of the intestinal epithelium into goblet cells (Figure 3G). Lastly, the intestine of chicks supplemented with Clostat + Sangrovit showed sloughing of the superficial epithelium with moderate metaplasia into goblet cells (Figure 3H), while chicks with supplemented with Gallipro showed severe desquamation of the superficial epithelium and metaplasia into goblet cells (Figure 3I).

## 4. Discussion

In the poultry industry, one of the most critical problems is necrotic enteritis (NE) [56]. Recently, antibiotics, antibacterial agents and probiotic agents have been becoming more common place as feed additives to enhance the health of animal, speed up growth and improve feed efficiency [57]. As a result, the supplementation of the diet with probiotic bacteria has increased [58]. Several studies have reported on the positive effects of the use of antibiotics and probiotics on the growth rate, feed utilization, feed efficiency and survival rate in broiler chickens challenged with *Clostridium spp.* [59,60]. However, strain selection, gene manipulation, strain combinations and probiotics combinations affect the degree of the positive effect attributed to the use of probiotics against the development of necrotic enteritis (NE) [61]. The best way of enhancing the action of probiotics seems to be by using multi-strain probiotics, which has a beneficial effect on the host by enhancing growth-promoting bacteria, combined with the viable antibiosis of pathogenic bacteria in the intestinal tract [62].

### 4.1. Growth Performance

In terms of growth, diet supplementation significant improved (*p* ≤ 0.05) the body weight gain (BWG), final body weight, production efficiency ratio (PER), feed conversion ratio (FCR) and survival rate of broiler chicks (days 0–35) during the overall experimental period improved groups compared to the chicks in the positive control group (Table 3). 

These results correlated with those reported by Khaksefidi and Ghoorchi [63], who supplemented the diets of chickens with 50 mg/kg of *Bacillus subtilis* and found that the body weight gain of the chicks increased significantly during the finisher period (days 22–42) compared to chickens fed control diets. Moreover, the supplementation of diets with probiotics significantly reduced the feed conversion ratio of broiler chickens (days 22–42) compared to the control diet group. Similarly, Patel et al. [61] found that the body weight gain and feed conversion ratio were significantly enhanced by the inclusion of probiotics (Protexin) at 100 g/ton in the diet, without any adverse effects on feed intake, mortality or carcass characteristics. Likewise, Anjum et al. [64] and Singh et al. [65] obtained similar results. The improvement of all the performance parameters may be due to the biological role of probiotics in the modification of intestinal pH, which benefits the bacterial population, improves nutrient absorption and increases the efficiency of feed utilization [66].

Diet supplementation was found to reduce the mortality rate of the broiler chicks, confirming the positive effects that supplementation with antibiotics, probiotics and phytobiotics have on mortality (Table 3). Our findings were similar to those reported by Abdel-Hafeez et al. [67]. Similarly, Riad et al. [68] found that the addition of probiotics in feed decreased the mortality rate in broiler chicks. An increased percentage of survival rate in broiler chicks has been previously ascribed to the inhibitory effects of these additives towards enteric pathogenic microorganisms via adjusting the intestinal pH [67].

### 4.2. Intestinal Histomorphometric Parameters

The mucosal architecture and villus structure are closely related to the absorption function of the small intestine [69]. However, few studies have compared the effect of types of probiotic on the intestine morphology of broiler chickens. Some researchers have indicated that the addition of probiotics resulted in an increased height of intestinal villus [70]. The effects of probiotic-supplemented diets on the histomorphometric parameters of the small intestine are listed in Table 4. In our study, the inclusion of probiotics in the diet significantly (*p* < 0.01) increased villus length (VL) and villus total area (VTA). We found a tendency for the villi width in the probiotics groups to increase, however, these differences were not statistically significant (*p* > 0.05). 

In the present study, the morphometric traits of the digestive tract were not significantly affected by diet supplementation with probiotics, apart from the small intestine weight (SIW), jejunum weight (JW), ileum weight (ILW) and lesion scores. Remarkably, we found a strong relationship between the small intestine weight and body weight [71]. In the present study, broiler chicks fed a diet supplemented with probiotics showed slight increases in SIW and ILW, which may be indicative of histological changes. We associated an increased villus height with an improved digestive and absorptive function of the intestine, due to an enlarged absorptive surface area, increased enzyme secretion and enhanced nutrient transport systems [72]. There is a strong correlation between increased villus height and the activation of intestinal villi function [73]. This suggests that the role of the villi is enhanced after the inclusion of probiotics in the diet. Moreover, improved inactive absorption of glucose and proline was previously reported in broiler chicks fed diets supplemented with *Lactobacilli*, *B. thermophilum* and *E. faecium* [70].

### 4.3. Blood Biochemical Parameters

Glucose is an important cellular source of energy and serves as a metabolic substrate [74]. The chicks that were administered probiotic-supplemented diets showed a significant increase (*p* ≤ 0.05) in their glucose levels and decreased ALT concentrations compared to the chicks in the positive control group. All other serum biochemical parameters did not show any significant differences between the different experimental groups. These findings correlate with those reported by Shareef and Al-Dabbagh [75], wherein serum glucose was found to be highest when probiotics were used against *Clostridium perfringens* infection in broiler chickens. Moreover, in a previous study, the concentration of certain serum biochemical parameters (total protein, lipids and albumin) in broiler chicks was not affected by probiotic supplementation [76]. Additionally, our results correlated with those reported by Al-Kassie et al. [77] and Ta et al. [78], since we did not find any significant differences between the chicks supplemented with probiotics and the control groups with regards to the blood composition (total protein, albumin and globulin). Moreover, Santoso et al. [79] found that the level of AST and ALT enzymes in the blood serum of broiler chickens was decreased by diet supplementation with probiotics. However, Hussein [80] found that probiotics (*Saccharomyces cerevisiae*) did not have any significant effect on serum AST and ALT activities in broiler chicks fed a supplemented diet compared to the control group. Diet supplementation with *Bacillus subtilis* and *E. faecium* results in normal liver function as a result of a significant decrease in the ALT and AST activities in the blood [81]. On the other hand, a significant increase in AST and ALT in the negative and positive control groups fed a basal diet could be used as an indicator of hepatocellular damage [82]. 

### 4.4. Liver and Intestinal Histopathological Signs

Supplementing diets with probiotics could help to prevent some of the harmful modifications caused by *Salmonella enterica* in the hepatocellular parenchyma [83]. NE can potentially lead to the degeneration and vacuolation of hepatocytes [84]. We found that the liver sections of chicks challenged with *Clostridium* were associated with several hepatocellular damages, including the congestion of portal blood vessels (Figure 1B) with numerous lymphocytic aggregations (Figure 1C), as well as edema and coagulative necrosis (Figure 1D). In contrast, the liver specimens of chicks fed supplemented diets showed an enhanced liver tissue structure against *Clostridium* challenge. The Sangrovit, Clostat + Sangrovit and Gallipro groups (Figure 1G, H and I) showed a normal hepatocellular structure with mild perivascular lymphocytic aggregation. The bacterial infection is the most important cause of lobular localization and may be a preamble to hepatocyte necrosis [85]. Moreover, the role of probiotics on the transfer of immune cells in the liver was reported by a previous study [86], which found that probiotic bacteria reduced the induction of monocytes and macrophages in the intestine and spleen of animals fed a supplemented compared with the controls. Finally, the supplementation of the diet with probiotics may enhance the enrollment of pro-inflammatory immune cells to systemic lymphoid tissues, including the liver and other organs [85]. 

Eeckhaut et al. [87] reported that probiotic *Butyricicoccus pullicaecorum* can prevent NE in the digestive tract of broiler chickens by reducing the number of potentially important pathogens in the caeca and ileum. In our recent study, the intestine samples from Gallipro- or Clostat-supplemented diets were examined and found to show a normal tissue architecture with normal intestinal villi (Figure 2F) and moderate metaplasia of the intestinal epithelium into goblet cells (Figure 3G), in addition to a negative control group (Figure 2A). Meanwhile, chicks challenged with *Clostridium* showed signs of injury to intestine tissue, including the degeneration and necrosis of intestinal villi, with extensive round cell aggregations (Figure 3B), wide hyperplasia of the villus epithelium, with a thickening and shortening of villi, together with metaplasia into goblet cells (Figure 3C), as well as a degeneration of the villous epithelium with metaplasia of the columnar epithelium into goblet cells (Figure 3D). These findings were in agreement with Wang et al. [56], who found that supplementing the diet of broiler chicks with *Lactobacillus plantarum* protected the intestinal structure from enterotoxigenic *Clostridium* infection by reducing irritation and preserving the integrity of the intestinal epithelial layer [88]. It is thus widely accepted that probiotics can prevent gut diseases by improving the immunity of the gut [89], promoting the development of gut histomorphology [90] and modifying the gut microbiota [91].

## 5. Conclusions

Using probiotic and phytobiotic compounds could be a useful strategy to preventing harmful effects of *C. perfringens* bacterium on the broiler performance, intestine, liver histopathological signs and some blood parameters alternative for using antibiotics. We suggest according to our study results that supplemented broiler diets with 0.5, 0.12 g Kg^−1^ of CloStat and Sangrovit Extra respectively, alone or in combined form promote growth and reduced the N.E. mortality rats.

## Figures and Tables

**Table 1:** Experimental groups and inclusion rates of feed additives used during the feeding trial period (day 0–35).

Item	Inclusion rate (g/kg)	Product resource
NC ^1^	‒	‒
PC ^2^	‒	‒
M ^3^	0.1	Maxus: 100 g of avilamycin (BIOFERM CZ, spol. sro.) per 1000 g.
CL^4^	0.5	CloStat: *Bacillus subtilis* (2 × 107 CFU/g) (KEMIN Ind., Valley Center, CA, USA) per 1 g.
S ^5^	0.12	Sangrovit Extra: Photobiotic compound (benzophenanthridine alkaloids, sanguinarine and protopine) (Albitalia s.r.L., Co., Milano, Italy)
CL + S ^6^	0.5 CL+ 0.12 S	CloStat + Sangrovit Extra
G ^7^	0.2	Gallipro Tech: A highly-selected strain (DSM17299) of *Bacillus subtilis* (4 × 10^9^ CFU/g DSM 17299) (Boege Alle Co., Hoersholm, Denmark)

^1^ Negative control = control with no additive or challenge; ^2^ PC = positive control; ^3^ M = 1 kg of the product contains 100 g *avilamycin*; ^4^ Clostat = *Bacillus subtilis* (2 × 10^7^ CFU/g); ^5^ *Sangrovit Extra* = 0.12 g/kg after *Clostridium* challenge; ^6^ CL + S = 0.5 g/kg CloStat + 0.12 g/kg Sangrovit Extra; ^7^ G = *Bacillus licheniformis* (1.5 × 10^11^ CFU/g) after *Clostridium* challenge.

**Table 2:** Composition of starter and finisher diets.

Ingredient	Treatment Period (0‒35) days	
	Starter (0–15)	Finisher (15–35)
Yellow corn	57.39	61.33
Soybean meal	27.00	22.80
Palm oil	2.20	2.80
Corn gluten meal	8.80	6.0
Wheat bran	0.00	3.0
DCP	2.30	2.09
Ground limestone	0.70	0.62
Choline chloride	0.05	0.05
DL-methionine	0.105	0.075
L-lysine	0.39	0.36
Salt	0.40	0.20
Threonine	0.17	0.17
V-M premix ^1^	0.50	0.50
Total	100	100
Analysis		
ME (kcal/kg)	3000	3050
Crude protein (%)	23.0	20.5
Non-phytate P (%)	0.48	0.44
Calcium (%)	0.96	0.88
Digestible lysine (%)	1.28	1.15
Digestible methionine (%)	0.60	0.54
Digestible sulfur amino acids (%)	0.95	0.86
Digestible threonine (%)	0.86	0.77

^1^ V-M premix; vitamin-mineral premix contains in the following per kg: vitamin A, 2,400,000 IU; vitamin D, 1,000,000 IU; vitamin E, 16,000 IU; vitamin K, 800 mg; vitamin B1, 600 mg; vitamin B2, 1600 mg; vitamin B6, 1000 mg; vitamin B12, 6 mg; niacin, 8000 mg; folic acid, 400 mg; pantothenic acid, 3000 mg; biotin 40 mg; antioxidant, 3000 mg; cobalt, 80 mg; copper, 2000 mg; iodine, 400; iron, 1200 mg; manganese, 18,000 mg; selenium, 60 mg; zinc, 14.000 mg.

**Table 3:** Effect of probiotic diet supplementation on growth performance and feed efficiency in broiler chicks challenged with *C. perfringens.*

			Treatments						
Parameters	NC	PC	M	CL	S	CL + S	G	SEM	Sig.
**IBW (g)**	36.8	36.7	36.9	36.8	36.8	36.9	36.8	0.04	NS
**FBW (g)**	1829.3 ^a^	1593.3 ^b^	1807.4 ^a^	1827.0 ^a^	1824.3 ^a^	1823.1 ^a^	1798.9 ^a^	2.121	***
**FI (g)**	2284.7	2325.6	2366.7	2353.2	2382.6	2379.9	2404.4	2.865	NS
**BWG (g)**	1424.6 ^a^	1134.0 ^b^	1384.3 ^a^	1379.0 ^a^	1418.8 ^a^	1386.5 ^a^	1350.8 ^a^	1.961	***
**FCR (g: g)**	1.60 ^c^	2.05 ^a^	1.71 ^b,c^	1.71 ^b,c^	1.68 ^b,c^	1.72 ^b^	1.78 ^b^	0.03	***
**PEF**	326.2 ^a^	215.6 ^c^	301.5 ^a,b^	305.0 ^a,b^	310.0 ^a,b^	307.6 ^a,b^	288.5 ^b^	2.76	***
**SR (%)**	100.0 ^a^	96.3 ^b^	98.1 ^a^	98.1 ^a^	98.1 ^a^	98.1 ^a^	98.1 ^a^	0.30	***

NC, negative control group; PC, positive control group; M, Maxus supplemented group; CL, CloStat supplemented group; S, Sangrovit Extra supplemented group; CL+S, CloStat+Sangrovit Extra supplemented group; G, Gallipro Tech supplemented group. IBW, initial body weight; FBW, final body weight; FI, feed intake; BWG, body weight gain; FCR, feed conversion ratio; PEF, protein efficiency ratio; SR, survival rate. SEM, mean values of standard error. Mean values of three replicates with deferent letter (^a, b, c^) in the same column are significantly deferent (*p* < 0.05). Sig., significance; N.S, non-significance; ***, significant at 0.001.

**Table 4:** Effect of probiotic diet supplementation on histomorphometric measurements in the intestine of broiler chicks challenged with *C. perfringens*.

	Treatments								
Parameters	NC	PC	M	CL	S	CL + S	G	SEM	Sig.
**Live body weight**	1815.9 ^a^	1593.3 ^b^	1807.4 ^a^	1827.0 ^a^	1824.3 ^a^	1823.1 ^a^	1798.9 ^a^	1.121	***
**Histomorphometry measurements**									
**VL (μm)**	629.98 ^a^	514.14 ^b^	576.82 ^a,b^	642.83 ^a^	632.40 ^a^	622.70 ^a^	627.28 ^a^	2.053	***
**VW (μm)**	71.433	60.858	71.433	69.426	72.323	73.565	73.141	3.759	NS
**VTA (mm^2^)**	0.142 ^a^	0.100 ^b^	0.135 ^a,b^	0.141 ^a^	0.144 ^a^	0.149 ^a^	0.150 ^a^	0.01	**
**Morphological measurements**									
**SIL (cm)**	211.3	191.6	202.9	206.3	194.6	201.7	205.8	2.14	NS
**DL (cm)**	15.66	18.29	17.81	17.00	17.51	16.75	15.97	0.89	NS
**CL (cm)**	19.03	17.89	17.76	17.36	23.37	17.61	17.45	2.17	NS
**JL (cm)**	42.13	39.41	39.72	41.51	41.14	41.69	41.41	0.88	NS
**ILL (cm)**	42.20	42.29	42.47	41.48	41.35	41.56	42.62	0.68	NS
**Relative SIW (%)**	0.63 ^a^	0.54 ^b^	0.54 ^b^	0.61 ^a,b^	0.56 ^a,b^	0.59 ^a,b^	0.54 ^b^	0.61	*
**Relative DW (%)**	0.051	0.066	0.055	0.053	0.068	0.057	0.052	0.10	NS
**Relative JW (%)**	0.202	0.161	0.151	0.175	0.169	0.169	0.156	0.24	NS
**Relative ILW (%)**	0.186 ^a^	0.144 ^b^	0.116 ^b^	0.136 ^b^	0.137 ^a,b^	0.123 ^b^	0.121 ^b^	0.09	**
**Relative CW (%)**	0.056	0.042	0.040	0.085	0.035	0.043	0.037	0.35	NS
**Lesion score**	0.00^b^	2.50 ^a^	0.67 ^b^	0.67 ^b^	0.33 ^b^	0.50 ^b^	0.67 ^b^	0.26	***

NC, negative control group; PC, positive control group; M, Maxus supplemented group; CL, CloStat supplemented group; S, Sangrovit Extra supplemented group; CL+ S, CloStat+Sangrovit Extra supplemented group; G, Gallipro Tech supplemented group. VL, villus length; VW, villus width; VTA, villus total area; SIL, small intestine length; SIW, small intestine weight; DL, duodenum length; DW, duodenum weight; JL, jejunum length; JW, jejunum weight; IL, ileum length; ILW, ileum weight; CL, caeca length; CW, caeca weight. (^a,b,c^) Significant differences (*p* < 0.001). Values within each column, means showing different letter of superscript were significantly different; N.S, non-significance; **, significant at 0.01; ***, significant at 0.001.

**Table 5:** Effect of probiotic diets supplementation on blood profile of broiler chicks challenged with *C. perfringens*.

			Treatments						
Parameters	NC	PC	M	CL	S	CL + S	G	SEM	Sig.
**TP (g/dL)**	2.3	2.0	2.2	2.3	2.2	2.2	2.4	0.081	NS
**ALB (g/dL)**	1.4	1.2	1.1	1.4	1.3	1.4	1.3	0.062	NS
**GLB (g/dL)**	0.9	0.8	1.0	0.9	0.9	0.8	1.1	0.088	NS
**Glu (mg/dL)**	134.5 ^a^	85.3 ^b^	124.9 ^a^	121.8 ^a,b^	134.8 ^a^	115.9 ^a,b^	130.0 ^a^	2.816	**
**ALT (IU/L)**	19.3 ^b^	41.8 ^a^	23.7 ^a,b^	21.2 ^b^	21.5 ^a,b^	27.0 ^a,b^	17.4 ^b^	1.433	*
**AST (IU/L)**	293.2	312.8	282.1	254.0	279.4	251.4	271.7	1.954	NS
**CHO**	76.2	76.5	71.6	76.7	72.5	83.9	77.3	3.268	NS
**TG**	34.9	46.4	44.4	47.2	39.9	46.9	50.9	2.043	NS

NC, negative control group; PC, positive control group; M, Maxus supplemented group; CL, CloStat supplemented group; S, Sangrovit Extra supplemented group; CL+ S, CloStat+Sangrovit Extra supplemented group; G, Gallipro Tech supplemented group. TP, total protein; ALB, albumin; GLB, globulin; Glu, glucose; ALT, alanine amine transferase; AST, alanine amine transferase; CHO, cholesterol; TG, total glyceride. ^a,b,c^ Significant differences *p* < 0.001). Values within each column, means showing different letter of superscript were significantly different; N.S, non-significance; *, significant at 0.05; **, significant at 0.01.
